# Different roles of axon guidance cues and patterned spontaneous activity in establishing receptive fields in the mouse superior colliculus

**DOI:** 10.3389/fncir.2014.00023

**Published:** 2014-03-26

**Authors:** Mingna Liu, Lupeng Wang, Jianhua Cang

**Affiliations:** ^1^Department of Neurobiology, Northwestern UniversityEvanston, IL, USA; ^2^Interdepartmental Neuroscience Program, Northwestern UniversityEvanston, IL, USA

**Keywords:** mouse visual system, superior colliculus, ephrins, retinal wave, on–off, direction selectivity, orientation selectivity

## Abstract

Visual neurons in the superior colliculus (SC) respond to both bright (On) and dark (Off) stimuli in their receptive fields. This receptive field property is due to proper convergence of On- and Off-centered retinal ganglion cells to their target cells in the SC. In this study, we have compared the receptive field structure of individual SC neurons in two lines of mutant mice that are deficient in retinotopic mapping: the ephrin-A knockouts that lack important retinocollicular axonal guidance cues and the nAChR-β2 knockouts that have altered activity-dependent refinement of retinocollicular projections. We find that even though the receptive fields are much larger in the ephrin-A knockouts, their On–Off overlap remains unchanged. These neurons also display normal level of selectivity for stimulus direction and orientation. In contrast, the On–Off overlap is disrupted in the β2 knockouts. Together with the previous finding of disrupted direction and orientation selectivity in the β2 knockout mice, our results indicate that molecular guidance cues and activity-dependent processes play different roles in the development of receptive field properties in the SC.

## INTRODUCTION

Neurons in the visual system respond to specific features of visual stimuli in their receptive fields (Kuffler, 1953; Hubel and Wiesel, 1962). The receptive field (RF) properties are determined by precise and selective connections in the brain and established by elaborative processes during development. For example, the RFs of neurons in many visual structures are organized into retinotopic maps, where neighboring neurons respond to neighboring locations in the visual space (Cang et al., 2005a, b; Wang and Burkhalter, 2007; Andermann et al., 2011; Marshel et al., 2011). The topographically precise projections from the retina to their targets, such as the superior colliculus (SC), are established by graded expression of molecular guidance cues such as EphAs and ephrin-As, and refined by activity-dependent processes driven by patterned spontaneous retinal activity (Cang and Feldheim, 2013). Disruption of either process could result in profound deficits in retinotopic mapping and subcortical visuomotor behaviors (Pfeiffenberger et al., 2006; Haustead et al., 2008; Wang et al., 2009). For the RFs of individual SC neurons, their structure and selectivity are disrupted when the patterns of retinal activity are altered during development (the nAChR-β2^-/-^ mice, Chandrasekaran et al., 2005; Wang et al., 2009). In contrast, the consequences of deleting ephrin-As or EphAs on collicular RF properties have not been studied, and as a result, the roles of molecular guidance cues and activity-dependent processes in the development of collicular RFs have not been directly compared.

In addition to spatial location, visual RFs are also characterized by their On and Off properties. The parallel On and Off pathways first diverge in the retina, with On- and Off-centered ganglion cells (RGCs) responding, respectively, to light increment and decrement, and a small population of On–Off RGCs responding to both (Kuffler, 1953). The On and Off pathways converge in the SC such that the On/Off subregions in the RFs of individual collicular neurons overlap almost completely (McIlwain and Buser, 1968; Cynader and Berman, 1972; Wang et al., 2010b). This On–Off convergence in the SC is believed to be important for detecting object salience, irrespective of its contrast (Knudsen, 2011).

In this study, we have compared the functions of guidance cues and activity-dependent processes in establishing the On–Off convergence in the SC. Surprisingly, we find that even though the RFs of SC neurons are much larger in the ephrin-A knockout mice, their On–Off overlap remains unchanged. These neurons also display normal level of direction and orientation selectivity. In contrast, the On-Off overlap is disrupted in the nAChR-β2^-/-^ mice. Together with the previous finding of disrupted direction and orientation selectivity in the β2^-/-^ mice, our results indicate that these two developmental processes play different roles in the development of RF properties in the SC.

## MATERIALS AND METHODS

### ANIMALS

Ephrin-A2/A5 double and A2/A3/A5 triple mutant mice were originally generated by the Feldheim Lab at University of California at Santa Cruz by crossing of each single line (Pfeiffenberger et al., 2006), and maintained in the animal facility at Northwestern University. Their genotypes were determined using the published protocols (Frisén et al., 1998; Feldheim et al., 2000; Cutforth et al., 2003). We previously studied the collicular RF properties in mice that lack the β2 subunit of nicotinic acetylcholine receptor (Wang et al., 2009) and in this study reanalyzed those data in the same way as for ephrin-A KOs (details below). Similarly, data from adult wild type C57BL/6 mice (Wang et al., 2010b) were reanalyzed for comparison. Both genders were used and all experiments were performed in accordance with protocols approved by Northwestern University Institutional Animal Care and Use Committee.

### *IN VIVO* ELECTROPHYSIOLOGY

Following our published procedures (Wang et al., 2010b), adult mice were anesthetized with urethane (1.2–1.3 g/kg in 10% saline solution, i.p.) and supplemented with chlorprothixene (10 mg/kg in 4 mg/ml water solution, i.m.). Atropine (0.3 mg/kg) and dexamethasone (2.0 mg/kg) were injected subcutaneously. Additional urethane (0.2–0.3 g/kg) was administered as needed. A tracheotomy was performed in some experiments and electrocardiograph leads were attached across the skin to monitor the heart rate continuously throughout the experiment. The animal’s temperature was monitored with a rectal thermal probe and maintained at 37°C through a feedback heater control module (FHC). Silicone oil was applied on the eyes to prevent from drying. A craniotomy (4–8 mm^2^) was performed on the left hemisphere to expose the brain for recording with 5–10 MΩ tungsten microelectrodes (FHC). The electrode was inserted vertically into the overlying cortex at a distance of 0.7–1.5 mm lateral of the midline suture and 0.2–0.8 mm anterior to the lambda suture. The identification of the SC surface followed our published procedure (Wang et al., 2010b). Only neurons within 300 μm below the SC surface were included in our analysis, corresponding to the superficial retinal recipient layers of the SC. Electrical signals were acquired using a System 3 workstation (Tucker Davis Technologies). Only one unit at a time was recorded in most cases. OpenSorter was used offline to remove occasional large electrical artifacts, or to sort two very different waveforms in a few cases. The animals were killed at the end of recordings by an overdose of euthanasia solution (150 mg/kg pentobarbital, in Euthasol, Virbac).

### VISUAL STIMULI AND DATA ANALYSIS

Visual stimuli were generated with customized Matlab programs (Niell and Stryker, 2008) using the Psychophysics Toolbox extensions (Brainard, 1997; Pelli, 1997). The stimuli were displayed on a flat panel CRT video monitor (40 cm × 30 cm, 60 Hz refresh rate, ∼35 cd/m^2^ mean luminance) placed 25 cm from the animal, and delivered to the eye contralateral to the recorded hemisphere while the ipsilateral eye was occluded. Stimulus sets included a blank condition in which the screen was at mean luminance. Responses to all such blank presentations were averaged to obtain the spontaneous firing rate.

To determine RF structures of SC neurons, 5° light squares were flashed at different locations on either a 13 × 13 or 11 × 11 grid with 5° spacing. The flashes stayed on for 500 ms on a gray background and off for 500 ms between stimuli, and were repeated for 4–6 times for each grid location in a pseudorandom sequence. Spontaneous firing was analyzed in the blank stimulus condition and the mean + 2 × SD of the spontaneous rate was calculated as threshold. The responses to flashing spots at each location were analyzed by counting spikes within a time window of 200 ms (starting from 50 ms after flash onset or offset) in each trial. The cell was considered responsive to On or Off at a given grid location, if there were more spikes than the threshold in at least 40% of the trials (Sarnaik et al., 2013). An On–Off overlap ratio was then calculated as the number of grids that showed both On and Off responses divided by the total number of responsive locations regardless of On or Off polarity. Additionally, correlation coefficients were calculated between On and Off responses over the entire grid from raw spike rates without thresholding (Wang et al., 2010b).

Full field and full contrast of drifting sinusoidal gratings were presented to probe selectivity for stimulus direction/orientation (0–360°, 12 steps at 30° spacing) and spatial frequency (0.01–0.32 cpd at six logarithmic steps; Wang et al., 2010a; Zhao et al., 2013a). Temporal frequency was fixed at 2 cycle/s. Each stimulus of given direction and spatial frequency (or a blank condition) was presented for 1.5 s in a pseudorandom order for 4–6 trials. The interval between stimuli was 0.5 s. The response to a particular stimulus condition, *R*, was obtained by averaging the number of spikes over the 1.5 s stimulus duration, across all trials and subtracting the spontaneous rate. The preferred direction was determined as the one that gave maximum response (*R*_pref_), averaging across all spatial frequencies. The preferred spatial frequency was the one that gave peak response at this direction. Responses across all directions at the preferred spatial frequency, *R*(*θ*), were used for further analysis. The depth of modulation was described using two parameters: (1) Direction Selectivity Index = *R*_pref_/(R_pref_ + R_opp_), where *R*_pref_ was the response at *θ*_pref_ and *R*_opp_ at *θ*_pref__+__π_ and (2) Orientation Selectivity Index = *R*′_pref_/(*R*′_pref_ + *R*_orth_), where *R*′_pref_ was the mean response of *R*_pref_ and *R*_opp_, *R*_orth_ was the mean response to the two directions orthogonal to *θ*_pref_. The tuning curves were fitted with a sum of two Gaussians centered at *θ*_pref_ and *θ*_pref__+__π_ using the *nlinfit* function in Matlab (Mathworks, Natick, MA, USA), and the tuning width was calculated as the half-width at half maximum of the fitted curve above the baseline. For mean tuning curves, each curve was normalized to the peak response and then aligned to the direction that elicited the maximum response.

### STATISTICAL ANALYSIS

All values were presented as mean ± SEM. Non-parametric tests that do not require any assumptions about the distribution of the data were used in all cases. Comparison of distributions was done using the two-sample Kolmogorov–Smirnov test (K–S test) and comparisons between means or medians of datasets were done using two-sample Mann–Whitney test. All statistical tests were evaluated at *α* = 5% probability of false positives. Two-sided statistical tests were performed. Statistical analyses and graphing were done in MATLAB and Prism (GraphPad Software Inc.).

## RESULTS

### DISRUPTED RECEPTIVE FIELDS IN SC NEURONS OF EPHRIN-A KO MICE

**FIGURE 1:**
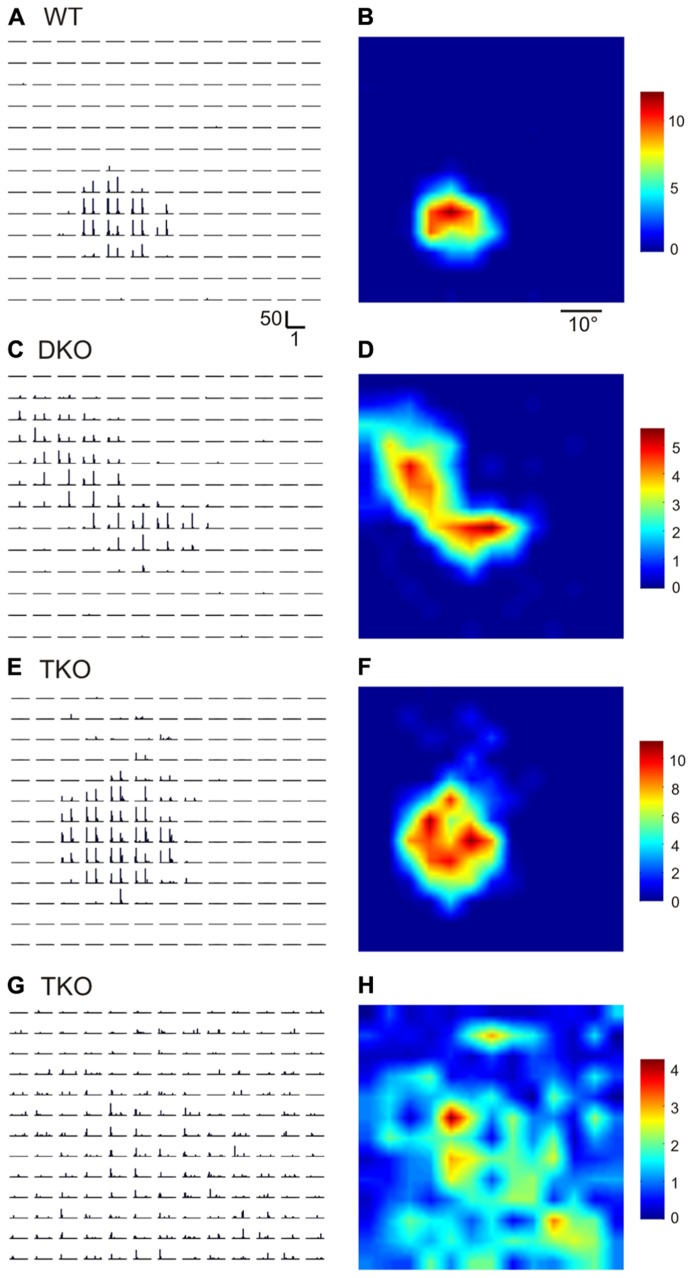
**Disrupted receptive field structures in the SC of ephrin-A knockout mice.** **(A,B)** Receptive field of a SC neuron in WT mouse. **A** shows peri-stimulus timing histograms (PSTH) in response to spots flashed at different locations on a 13 × 13 grid in visual space. Scale bars are 50 spikes/s (y-axis, for firing rate in each 50 ms bin) and 1 s (x-axis). Both On and Off responses were evoked within the receptive field, as indicated by the two peaks in individual PSTHs. The receptive field structure determined by the PSTHs is shown in **B** in a color scale (right, in spikes/s, for mean firing rate in the 1 s stimulus duration). **(C–H)** Example receptive fields of SC neurons in ephrin-A double (DKO) and triple KO (TKO) mice.

Ephrin-A2, A3, and A5 are the three main ephrin-As expressed in the developing visual system in mice. In this study, we used single unit recording to characterize the RFs of SC neurons in mice lacking all three of the ephrins (triple knockouts, TKO), or two of them (A2 and A5, double knockouts, DKO). To determine RF structure, we flashed small spots (5°×5°) at different positions in the visual field (Wang et al., 2010b). Compared to wild type (WT) SC neurons, which only responded to flashes within a small region in the visual space (**Figures 1A,B**), the RFs of many SC neurons in the ephrin-A KOs were much larger. By visual inspection, some neurons in the mutant mice had multiple patches within their RFs (e.g., **Figures 1C,D**; *n* = 36 out of 85 cells in DKO and 11/33 cells in TKO), while others had single patches that still appeared larger than in WT (**Figures 1E,F**; *n* = 16/85 in DKO and 8/33 in TKO). A small number of cells even had very diffuse RFs that expanded across almost the entire stimulus monitor (**Figures 1G,H**; *n* = 8/85 in DKO and 3/33 in TKO).

**FIGURE 2:**
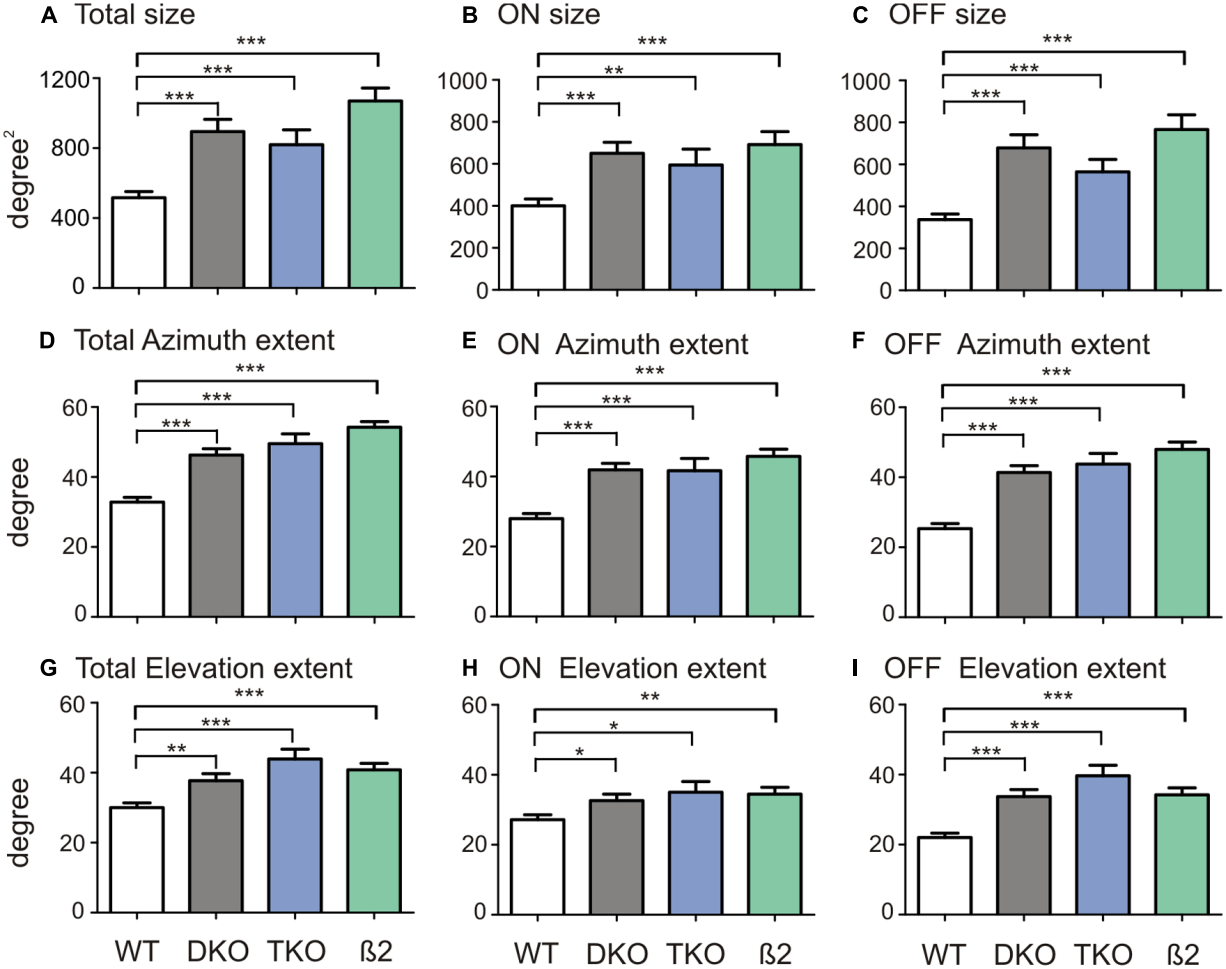
**Quantification of SC receptive field structures.** **(A)** Comparison of receptive field size between SC neurons in WT mice and ephrin-A DKOs, TKOs, and nAChR-β2 KOs. **(B)** Comparison of the ON subregion size between groups. **(C)** Comparison of the OFF subregion size between groups. **(D–F) **Comparison of receptive field extent along the azimuth axis **(D)** and that of the On **(E)** and Off subregions **(F)** between groups. **(G–I) **Comparison of receptive field extent along the elevation axis **(G)** and that of the On **(H)** and Off **(I)** between groups. All error bars represent SEM, and *p < 0.05, **p < 0.01, and ***p < 0.001.

Because the RFs of many neurons in the ephrin-A KOs had irregular shapes, they could not be fitted into 2-d Gaussians as we previous did in WTs to quantify RF size (Wang et al., 2010b). We thus simply counted the number of grid positions where visual responses were evoked by the flashing spots (see Materials and Methods for details). The RFs of SC neurons in ephrin-A KOs (DKOs: mean = 894.7 ± 69.8°degree^2^, median = 750.0 degree^2^, *n* = 85; TKOs: mean = 819.7 ± 84.5 degree^2^, median = 700.0 degree^2^, *n* = 33) indeed occupied much larger area compared to those in the WT (mean = 516.3 ± 35.5 degree^2^, median = 400.0 degree^2^, *n* = 101; *p* < 0.0001 Mann–Whitney test; **Figure 2A**). The RFs were similarly enlarged in the DKOs and TKOs, consistent with the notion that ephrin-A2 and A5 are the most important cues in retinocollicular mapping (Feldheim et al., 2000; Pfeiffenberger et al., 2006). We also examined whether the disruption was restricted to the azimuth axis of the visual space since ephrin-As mediate the mapping of retinocollicular axons along the naso-temporal axis (Cang and Feldheim, 2013). We calculated the azimuth and elevation extent covered by individual RFs and found that they were enlarged along both axes in the ephrin-A KOs, though the disruption appeared more severe along the azimuth axis (**Figures 2D,G**. Azimuth: WT, mean = 32.8 ± 1.4°, median = 30.0°, *n* = 101; DKO, mean = 46.3 ± 1.8°, median = 50.0°, *n* = 85, *p* < 0.0001; TKO, mean = 49.6 ± 2.8°, median = 60.0°, *n* = 33, *p* < 0.0001; Elevation: WT, mean = 30.0 ± 1.4°, median = 25.0°, *n* = 101; DKO, mean = 37.7 ± 2.0°, median = 35.0°, *n* = 85, *p* < 0.01; TKO, mean = 43.9 ± 2.8°, median = 45.0°, *n* = 33, *p* < 0.0001; Mann–Whitney test). These results thus demonstrate that axonal guidance cues are needed, either directly or indirectly, for the development of spatially compact RFs of SC neurons.

### NORMAL ON–OFF OVERLAP IN EPHRIN-A KOs DESPITE DISRUPTED RECEPTIVE FIELDS

Most visual neurons in WT SC respond to both bright (On) and dark (Off) stimuli and the ON and Off regions overlap spatially within their RFs (Wang et al., 2010b). Such On–Off overlap is a conserved feature in the SC of all the species studied so far (McIlwain and Buser, 1968; Cynader and Berman, 1972; Rhoades and Chalupa, 1977; Prevost et al., 2007), and is the basis of SC’s ability to detect salient visual events irrespective of contrast. We thus investigated whether such a fine scale feature of RF organization is disrupted in the ephrin-A KOs.

We first divided On and Off responses and analyzed their subregion size separately. In the ephrin-A KOs, both ON (DKO: mean = 650.0 ± 52.7 degree^2^, median = 500.0 degree^2^, *n* = 85, *p* < 0.0001; TKO: mean = 594.7 ± 75.6 degree^2^, median = 500.0 degree^2^, *n* = 33, *p* < 0.01; Mann–Whitney test) and Off subregions (DKO: mean = 678.8 ± 62.2 degree^2^, median = 525.0 degree^2^, *n* = 85, *p* < 0.0001; TKO: mean = 564.4 ± 59.7 degree^2^, median = 500.0 degree^2^, *n* = 33, *p* < 0.0001) were bigger than in WTs (ON, mean = 400.5 ± 32.2 degree^2^, median = 300.0 degree^2^, *n* = 101; Off, mean = 336.9 ± 26.6 degree^2^, median = 300.0 degree^2^, *n* = 101). The subfield expansion in ephrin-A KOs was along both elevation and azimuth axes, consistent with their enlarged RF in general (**Figure 2**).

**FIGURE 3:**
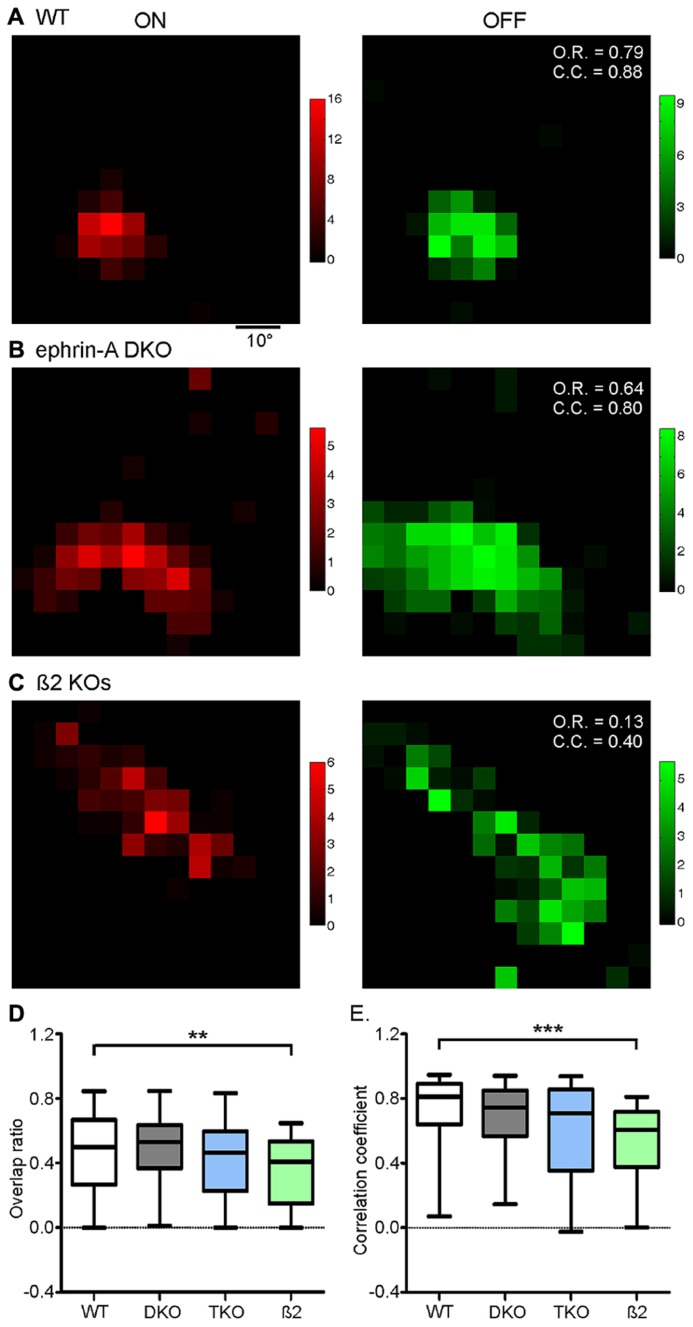
**On–Off overlap is disrupted in nAChR-β2 KOs, but not in ephrin-A KOs.** **(A)** On (red) and Off (green) responses of a WT SC neuron, showing substantial On–Off overlap. Color scales represent evoked responses in spikes/s during the 500 ms duration of stimulus presentation. The calculated values of overlap ratio (O.R.) and correlation coefficient (C.C.) are listed at the upper right corner of the “Off” plot. **(B)** On and Off responses of an example neuron in ephrin-A DKO mice. **(C)** Responses of an example neuron in nAChR-β2 subunit knockout. **(D)** Comparison of On–Off overlap ratio between genotypes, with only β2 KOs showing a significant disruption comparing to the WT. **(E)** Comparison of On–Off correlation coefficient between genotypes. Panel **D** and **E** are box plots with ends of each plot representing 5th and 95th percentiles. **p < 0.01 and ***p < 0.001.

We next quantified On–Off overlap using an overlap ratio for each neuron, calculated as the ratio of the number of grids that showed both On and Off responses over the total number of responsive locations regardless of On or Off polarity. The overlap ratio ranges from 0 to 1, with a value of 1 indicating complete On–Off overlap, and 0 no overlap (or the cell only has one subfield). Surprisingly, despite the disruption of RF size, the On–Off overlap ratios in the ephrin-A KOs (e.g., **Figure 3B**; DKO: mean = 0.50 ± 0.03, median = 0.53, *n* = 85; TKO: mean = 0.42 ± 0.05, median = 0.46, *n* = 33) were similar to that in WT (e.g., **Figure 3A**; mean = 0.46 ± 0.03, median = 0.50, *n* = 101; *p* = 0.47 and *p* = 0.94, respectively, K–S test; **Figure 3D**). We also quantified the On–Off overlap by calculating the correlation coefficient, which takes into account response magnitude at each stimulus location. Again, the On–Off correlations did not show a significant difference between ephrin-A KOs (**Figure 3E**; DKO: mean = 0.68 ± 0.03, median = 0.74, *n* = 85; TKO: mean = 0.61 ± 0.05, median = 0.71, *n* = 33) and WT (mean = 0.71 ± 0.03, median = 0.81, *n* = 100; *p* = 0.12 and 0.06, respectively, K–S test). In other words, the On–Off overlap in collicular RFs is largely maintained in the absence of ephrin-A guidance cues.

### DISRUPTED ON–OFF OVERLAP IN nAChR-β2 KOs

The above results prompted us to ask what factors, if not ephrin-As, might be required for the development of On–Off convergence in the SC. Previous studies showed that spontaneous retinal waves drive the refinement of retinocollicular map (McLaughlin et al., 2003; Chandrasekaran et al., 2005; Xu et al., 2011). In mice that lack the β2 subunit nicotinic ACh Receptor (β2 KOs), the patterns of retinal waves are disrupted (Bansal et al., 2000; McLaughlin et al., 2003; Sun et al., 2008; Stafford et al., 2009) and the RF of SC neurons were enlarged (Chandrasekaran et al., 2005; Wang et al., 2009). We thus analyzed the On–Off overlap in these mice. The On and Off subregions in β2 KOs were similarly large as in the ephrin-A KOs (**Figure 2**). But importantly, unlike in the ephrin-A KOs, the On–Off overlap in β2 KOs, both by overlap ratio (mean = 0.36 ± 0.03, median = 0.41, *n* = 59; *p* = 0.01, K–S test) and correlation coefficient (mean = 0.53 ± 0.03, median = 0.61, *n* = 59, *p* < 0.0001), was significantly reduced (**Figures 3C–E**).

Together, these results indicate that ephrin-As are not required for establishing the overlapped On–Off subfields of mouse SC neurons, but instead the activity-dependent refinement process is necessary for its development.

### NORMAL RESPONSES TO DRIFTING GRATINGS IN EPHRIN-A KOs

**FIGURE 4:**
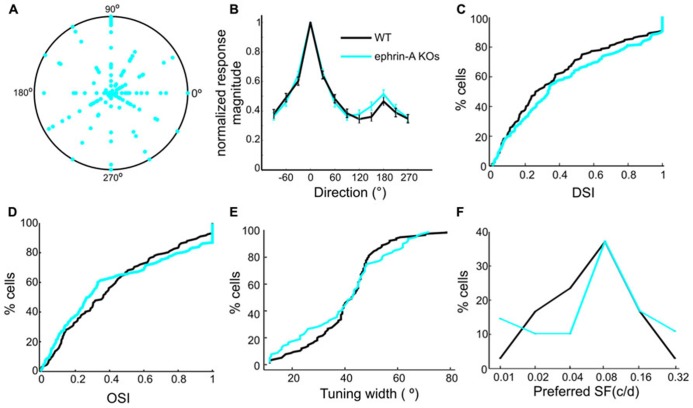
**Normal responses to drifting gratings in ephrin-A KOs.** **(A)** Polar plot of direction selectivity index (DSI, radii from origin) and preferred directions (angles). Each dot is from one cell. The outer circle represents DSI value of 1. **(B)** Normalized direction tuning curve of ephrin-A KOs and WT SC neurons, plotting the mean and SEM of normalized responses at each direction. **(C)** Cumulative distribution of DSI in the two genotypes (ephrin-A KOs, mean = 0.43 ± 0.03, median = 0.33, *n* = 137; WT, mean = 0.38 ± 0.03, median = 0.27, *n* = 132; *p* = 0.42, K–S test). **(D)** Cumulative distribution of OSI in the two genotypes (ephrinA-KOs, mean = 0.41 ± 0.03, median = 0.28, *n* = 137; WT, mean = 0.41 ± 0.03, median = 0.36, *n* = 132; *p* = 0.15, K–S test). **(E)** Cumulative distribution of tuning width (*p* = 0.41, K–S test). **(F)** Distribution of preferred spatial frequency. Similar percentage of cells in the two genotypes preferred 0.08 cpd and 0.16 cpd.

In addition to static contrast changes, SC neurons are also sensitive to moving stimuli (Wang et al., 2010b). We thus examined the tuning properties of SC neurons in the ephrin-A KO mice in response to drifting gratings. Recordings from the DKOs and TKOs were combined together since no difference was seen between them. Remarkably, many SC neurons in the KOs were selective for stimulus direction or orientation, just like in WT. Across the population, the preferred directions did not show any bias towards certain angles (**Figure 4A**), similar to those in WT SC (Wang et al., 2010b). This result is clearly different from that of the β2 KOs, in which fewer SC neurons are tuned to horizontal motion (Wang et al., 2009). The degree of direction/orientation selectivity was also normal in the ephrin-A KO mice, both by averaged tuning curves (**Figure 4B**) and the distribution of direction and orientation selectivity index (**Figures 4C,D**). Consistently, the orientation tuning width in the ephrin-A KOs (mean = 39.8 ± 1.9°, median = 42.8°, *n* = 78) was also similar (**Figure 4E**, *p* = 0.41, K–S test) to that in the WT mice (mean = 40.8 ± 1.2°, median = 42.5°, *n* = 115). Furthermore, no change of response linearity as determined by F1/F0 ratio (Wang et al., 2010b) was found between the ephrin-A KOs (mean = 0.62 ± 0.04, median = 0.51, *n* = 137) and WT (mean = 0.64 ± 0.05, median = 0.41, *n* = 132; *p* = 0.36, K–S test). Finally, although the distribution of preferred spatial frequency was statistically different between the ephrin-A KOs and WTs (*p* < 0.001, *χ*^2^ test), most neurons preferred 0.04, 0.08 and 0.16 cpd in both genotypes (**Figure 4F**).

These results thus indicate that the removal of ephrin-As has little effect on the orientation and direction selectivities of individual SC neurons, despite their altered RF structures. Together with our previous findings that the SC neurons in the β2 KOs display axis-specific disruption of direction and orientation selectivity (Wang et al., 2009), our results demonstrate that axonal guidance cues and activity-dependent processes play different roles in the development of visual response properties in SC neurons.

## DISCUSSION

In this study, we have examined the RF structure of SC neurons in two lines of mutant mice that are deficient in retinocollicular mapping, the ephrin-A KOs and the nAChR-β2 KOs that have altered retinal waves. Our results reveal that even though the collicular RFs are similarly enlarged in the two mutants, the On/Off overlap within the RF is maintained in the ephrin-A KOs but disrupted in the β2 KOs. During development, retinal axons are guided to their target cells in the SC by graded guidance cues such as ephrin-As and the remaining aberrant projections are then eliminated through activity-dependent processes driven by spontaneous retinal waves (Eglen et al., 2003; Grimbert and Cang, 2012). As a result, only ganglion cells from a small patch of the retina, both On and Off, are left innervating individual collicular neurons, giving rise to spatially compact RFs with overlapping On and Off subregions. In the absence of ephrin-As, the nasal-temporal retinotopic information is lost and RGCs from distant regions of the retina can terminate onto the same SC neurons. Our results indicate that nearby On and Off neurons still co-terminate in the ephrin-A KOs, presumably driven by largely normal retinal waves in these mice, which display WT level of correlation within small distances (Pfeiffenberger et al., 2005). On the other hand, in the β2 KOs, this process is disrupted, leading to some nearby On and Off RGCs no longer innervating the same SC neurons, due to either compromised elimination or aberrant expansion of axonal terminals in these animals (Dhande et al., 2011).

The exact patterns of retinal waves in the β2 KOs have been controversial. Whereas earlier studies showed that there were no correlated activities in the RGCs of these mice (Bansal et al., 2000; McLaughlin et al., 2003), more recent studies revealed that they did display retinal waves (Sun et al., 2008; Stafford et al., 2009), which appeared to correlate RGCs over broader distances (about twice as far as in WT retinas) and with a weaker intensity (about half the WT peak amplitude; Stafford et al., 2009). Importantly, whether there are larger waves or no waves, the information for differentiating RGCs that are immediately next to each other and those that are further apart is compromised, which could then lead to disrupted retinotopic mapping and On/Off convergence.

Our explanation of the On/Off phenotypes in ephrin-A KOs and WT mice assumes that On and Off RGCs are similarly correlated in retinal waves during the time of retinocollicular development. At postnatal day 12, when retinocollicular mapping has reached the mature level (Dhande et al., 2011) and retinal waves are already mediated by glutamatergic transmission, On and Off RGCs fire with a temporal offset during the waves (Kerschensteiner and Wong, 2008). Such an asynchronous pattern was not seen earlier during development when the waves are cholinergic (Kerschensteiner and Wong, 2008). It is thus highly likely that neighboring On and Off RGCs fire synchronously when retinocollicular connections are established, which would lead to On/Off convergence and consequently On–Off overlap in the RF of SC neurons.

SC neurons’ selectivity for stimulus orientation and direction is also different between ephrin-A KOs and β2 KOs. The mechanism of SC selectivity is still unclear. On the one hand, it could be inherited from the retina, given a substantial population of RGCs are direction/orientation selective in mice (Elstrott et al., 2008; Huberman et al., 2009; Zhao et al., 2013b). The direction selective RGCs (DSGCs), including On, Off, and On–Off subtypes, are tuned to motions of unique directions, such as the four cardinal directions for On–Off DSGCs (Elstrott et al., 2008; Kim et al., 2008; Huberman et al., 2009). These RGCs could converge onto SC neurons and give rise to a preference for certain directions or axes of motion. The results that the selectivity is largely normal in ephrin-A KOs but disrupted along the azimuthal axis in β2 KOs thus suggest that the activity-dependent refinement could be important for converging different subtypes of DSGCs, just as in converging On and Off-centered RGCs in creating overlapped RFs. On the other hand, SC direction/orientation selectivity could result from circuits within the colliculus, such as inhibition from local GABAergic interneurons. These interneurons are known to shape many aspects of SC responses (Binns and Salt, 1997), although their roles in SC selectivity have not been investigated. In such a scenario, our results would indicate that molecular guidance cues such as ephrin-As are not critical, while the activity-dependent processes are more important, in establishing these intracollicular connections.

## AUTHOR CONTRIBUTIONS

Mingna Liu and Lupeng Wang performed the experiments. Mingna Liu, Lupeng Wang and Jianhua Cang designed the study, analyzed data and wrote the article. The authors declare no competing financial interests.

## Conflict of Interest Statement

The authors declare that the research was conducted in the absence of any commercial or financial relationships that could be construed as a potential conflict of interest.
